# Low to moderate dose ^137^Cs (γ) radiation promotes M2 type macrophage skewing and reduces atherosclerotic plaque CD68+ cell content in ApoE^(−/−)^ mice

**DOI:** 10.1038/s41598-024-63084-x

**Published:** 2024-05-30

**Authors:** N. Rey, T. Ebrahimian, C. Gloaguen, D. Kereselidze, E. Christelle, C. Brizais, F. Bachelot, G. Riazi, V. Monceau, C. Demarquay, I. Garali Zineddine, D. Klokov, S. Lehoux, Teni G. Ebrahimian

**Affiliations:** 1 Present Address: Institut de Radioprotection et de Sûreté Nucléaire, Laboratoire de Radiotoxicologie et de Radiobiologie Expérimentale, 92262 Fontenay-Aux Roses, France; 2 Department of Medicine, Lady Davis Institute for Biomedical Research, McGill University, Montreal, Canada

**Keywords:** Atherosclerosis, Atherosclerosis

## Abstract

The effects of low doses of ionizing radiation on atherosclerosis remain uncertain, particularly as regards the generation of pro- or anti-inflammatory responses, and the time scale at which such effects can occur following irradiation. To explore these phenomena, we exposed atheroprone ApoE^(−/−)^ mice to a single dose of 0, 0.05, 0.5 or 1 Gy of ^137^Cs (γ) administered at a 10.35 mGy min^−1^ dose rate and evaluated short-term (1–10 days) and long-term consequences (100 days). Bone marrow-derived macrophages were derived from mice 1 day after exposure. Irradiation was associated with a significant skewing of M0 and M2 polarized macrophages towards the M2 phenotype, as demonstrated by an increased mRNA expression of Retnla, Arg1, and Chil3 in cells from mice exposed to 0.5 or 1 Gy compared with non-irradiated animals. Minimal effects were noted in M1 cells or M1 marker mRNA. Concurrently, we observed a reduced secretion of IL-1β but enhanced IL-10 release from M0 and M2 macrophages. Effects of irradiation on circulating monocytes were most marked at day 10 post-exposure, when the 1 Gy dose was associated with enhanced numbers of both Ly6C^High^ and Ly6^Low^ cells. By day 100, levels of circulating monocytes in irradiated and non-irradiated mice were equivalent, but anti-inflammatory Ly6C^Low^ monocytes were significantly increased in the spleen of mice exposed to 0.05 or 1 Gy. Long term exposures did not affect atherosclerotic plaque size or lipid content, as determined by Oil red O staining, whatever the dose applied. Similarly, irradiation did not affect atherosclerotic plaque collagen or smooth muscle cell content. However, we found that lesion CD68+ cell content tended to decrease with rising doses of radioactivity exposure, culminating in a significant reduction of plaque macrophage content at 1 Gy. Taken together, our results show that short- and long-term exposures to low to moderate doses of ionizing radiation drive an anti-inflammatory response, skewing bone marrow-derived macrophages towards an IL-10-secreting M2 phenotype and decreasing plaque macrophage content. These results suggest a low-grade athero-protective effect of low and moderate doses of ionizing radiation.

## Introduction

Thousands of people, many living in areas close to Chernobyl or to the Fukushima power plant, are regularly exposed to low doses of γ radiation, as defined by the United Nations Scientific Committee on the Effects of Atomic Radiation (UNSCEAR report 2012, annex A). Understanding the effects of such low to moderate doses of ionizing radiation (LMDIR) on atherosclerosis is of critical importance. Atherosclerosis is a chronic inflammatory disease of medium and large arteries that is associated with myocardial infarction and stroke^1^. Cardiovascular diseases are the leading cause of death globally. It has been reported that cardiovascular diseases are aggravated by exposure to high ionizing radiations^2–7^***.*** However, extrapolation through the linear no-threshold model does not yield reliable relationships between low dose exposure and cardiovascular diseases^8^ and limits in statistical power preclude epidemiological risk assessment for doses under 0.5 Gy^9^. Understanding the risk associated with a given dose of exposure is further complicated by recent studies showing that effects caused by high doses are different from those caused by low doses and are influenced by the applied dose-rate. Mitchell et al.^10^ showed that effects of low doses were nonlinear in ApoE^(−/−)^ mice, where low doses given at low dose rate were atheroprotective, but high dose rates produced detrimental effects. Another study confirmed that atherosclerotic lesion area and composition depended on both the dose and dose-rate applied, with effects ranging from neutral (low dose and low dose-rate) to deleterious (higher dose and/or dose-rate)^11^. Le Gallic et al.^12^ demonstrated that exposure to chronic LMDIR enhances plaque stability in ApoE^(−/−)^ mice. These results confirm the modulatory effects of LMDIR in inflammatory conditions^13^. Additionally, a clinical study demonstrated that repeated LMDIR with a final dose of 3 Gy could have beneficial long term effects on painful skeletal disorder^14^. Hence, exposure to high doses of ionizing radiation is reported to have long term effects on human health, including cardiovascular disease, but at present the possible effects of LMDIR on atherosclerosis are still unclear^15^.

The innate immune system is essential for the development and progression of atherosclerosis. Plaque formation is driven by the entry of oxidized low density lipoproteins into the intima of arteries, triggering a pro-inflammatory reaction leading to the transmigration of monocytes^1^. Two subsets of circulating monocytes are identified in mice: pro-inflammatory Ly6C^High^ and patrolling Ly6C^Low^^16–18^. Ly6C^High^ cells express high levels of the chemokine receptor CC-chemokine receptor 2 (CCR2)^19,20^, are recruited from the bone marrow and the spleen, and tend to give rise to M1 type pro-atherogenic macrophages^21^. They produce pro-inflammatory cytokines such as TNF-α, IL-18, IL-12, IFN-γ or IL-1. In comparison, Ly6C^Low^ monocytes are involved in wound repair and tissue remodeling^22^. They preferentially express CX3C-chemokine receptor 1 (CX3CR1)^23^ and generally give rise to M2 type athero-protective macrophages^22,24^. They produce anti-inflammatory cytokines such as TGF-β or IL-10^25–27^. In atherosclerotic lesions, monocyte-derived macrophages take up oxidized lipids and become foam cells that accumulate in the vessel wall. Build-up of foam cells encroaches onto the vascular lumen and renders lesions prone to rupture, associated with clinical events. The inflammatory milieu also attracts different immune cell types such as T cells, which can also be pro- or anti-inflammatory^28,29^, as well as smooth muscle cells that produce collagen and thus reduce plaque vulnerability to rupture.

Currently, the effects of low and moderate doses of ionizing radiation on atherosclerosis are not well understood. The aim of this study is to investigate short term effects of LMDIR on macrophage polarization, gene expression and cytokine production, as well as to evaluate potential long-term effects of LMDIR on atherosclerotic plaque size and composition. We hypothesize that LMDIR impacts monocyte and macrophage polarization, influencing atherosclerotic lesion growth and stability.

## Materials and methods

This study is reported in accordance with ARRIVE guidelines.

### Animals

All experiments and procedures were carried out in accordance with the Guide for the Care and Use of Laboratory Animals as published by the French regulations for animal experiments (Ministry of Agriculture Order No. B92-032-01, 2006) with European Directives (86/609/CEE), and approved by the local ethical committee of the Institute for Radiological Protection and Nuclear Safety (permit number P15-06).

Fourteen- to 16-week-old ApoE^(−/−)^ mice were purchased from Charles River and were maintained in our animal facility during 1 week prior to experiments. Experiments were evaluated and approved by an internal animal ethical committee. Apolipoprotein E acts as the main ligand mediating removal of cholesterol enriched chylomicron and very low-density lipoprotein remnants from the blood stream and plays an important role in lipoprotein metabolism. ApoE^(−/−)^ mice develop atherosclerosis even when fed a normal chow diet. The morphological features of early-stage lesions in ApoE^(−/−)^ mice are very similar to those found in humans^30^. Animals were maintained in a specific-pathogen-free environment and monitored daily.

Mice were exposed to a single LMDIR at 0.05, 0.5, or 1.0 Gy of γ rays from an external source of ^137^Cs with a 10.35 mGy/min dose rate. The animals were sacrificed 1, 10, or 100 days post-irradiation. Short term studies were designed to evaluate the effects of irradiation on bone marrow-derived primary cells, whereas the long exposure served to assess atherosclerotic lesions in mice. Blood (obtained by cardiac puncture) was collected at the end of each experiment under ketamine/xylazine anesthesia (300 µL IP) and depleted of erythrocytes with ACK lysis buffer for flow cytometry analysis. Following cervical dislocation, bone marrow cells were collected and differentiated as described below. Spleens were collected at days 10 and 100 following irradiation for flow cytometry. The heart was collected at day 100 for atherosclerotic lesion evaluation.

### Isolation and differentiation of bone marrow-derived macrophages (BMDM)

For cell isolation, femurs were isolated and placed in a sterile petri dish containing sterile RPMI+ cell culture medium (RPMI 1640, 10% fetal bovine serum and 1% streptomycin/penicillin). The bone marrow was extracted by flushing with a 25-G needle, and cells were filtered with a 70 µm filter and centrifuged 10 min at 500*g*. Cells were counted, distributed into 6-well cell culture plates at a concentration of 10^6^ cells ml^−1^, and incubated at 37 °C in 5% CO_2_/95% air for 1 h prior to medium change with RPMI+ containing 50 ng ml^−1^ of macrophage colony-stimulating factor (M-CSF) (Preprotech ref #315-02). Cells were maintained in culture for 5 days, with change of medium every 48 h to remove non-adherent cells. On the fifth day, cells were maintained in the same medium (M0) or polarized into M1 with IFNγ (50 ng ml^−1^) (Preprotech ref #315-05) or into M2 with IL-4 (10 ng ml^−1^) (Preprotech ref #214-14) for 24 h.

### Isolation of splenocytes

Splenocytes were obtained from spleens that were crushed and then passed through a 70 µm filter.

### Flow cytometry

All cells (10^6^ cells per tube) were first incubated with a FcR blocking reagent (# 130-059-901- Mylteni) for 15 min at 4 °C. BMDMs were then labeled with a mix of CD11b (PeCy7 clone M1/70 #11-0112-82 Invitrogen), F4/80 (PE clone 30 F11 #12-4801-82 Invitrogen), and CD206 (APC #46879 Invitrogen) antibodies. Circulating cells and splenocytes were labeled with a mix of 6 antibodies: CD115 (PE/Cy7 anti-mouse clone AF598 Biolegend #135524), CD11b (Efluor450 Clone M1/70 # 48-9668-80, Invitrogen), Ly6C (APC, clone RB6-8C5, # 17-5931-82), Ly6 (Gr-1) (FITC, clone 1A8-Ly6g #11-0112), Cx3CR1 (Percp/Cy5.5 anti-mouse clone 5A011F11 #149010 Biolegend), and CCR2 (PE #150610, clone 5A203G11 Biolegend). After, all cells were washed twice with PBS containing 2% FBS, centrifuged at 500*g*, and kept on ice until cytometry. Fluorescence was measured using a FACSCanto II (BD Biosciences) and analyzed with Flowjo Software. Compensation beads were used for color compensation, and FMO samples were used as negative control.

### Atherosclerotic plaque evaluation

The heart was isolated and removed from mice sacrificed 100 days post irradiation, fixed in 4% paraformaldehyde for 2 h, and placed in sucrose solution overnight before being embedded in OCT medium. Cryosections of 7 µm thickness were cut through the aortic sinus for histological and immunohistochemical analysis. Five to ten sections per animal were assessed for each stain and quantified by two independent researchers blinded to the animal exposure status. Slides were stained with Oil Red O (Sigma-Aldrich) to evaluate plaque lipid content, and with picro-sirius red (Sigma Aldrich) to evaluate plaque collagen content. Foam cell and vascular smooth muscle cell plaque content were determined by immunofluorescent staining with PE anti-mouse CD68 (diluted 1:100) (Biolegend clone FA-11) and FITC anti-α-smooth muscle actin (diluted 1:250) (Sigma-Aldrich F3777) antibodies, respectively. Lymphocyte content was assessed with anti-human CD3 (diluted 1:100) (DAKO, #A0452) followed by goat-anti rabbit AF488 (diluted 1:400) antibodies. Nuclei were stained with DAPI. Images were acquired using Axioscan. Quantification of the mean lesion size and positive stain area of plaque components calculated as a percentage of total lesion area were done using Histolab software.

### Cytokine secretion assay

Cell culture supernatants of polarized macrophages were harvested and stored at − 80 °C. IL-10 (M1000B), TGF-β (MB100B), TNF-α (MTA00B), IL-6 (M6000B), and IL-1β/IL-1F2 (MLB00C) levels were determined by ELISA (R&D Systems) according to manufacturer’s instructions. Briefly, 50 µl of undiluted supernatant was incubated in flat transparent 96 well plate pre-coated with primary antibody for 2 h, wells were washed 4–5 time, and the secondary antibody was added and incubated for another 2 h before another wash. Samples were revealed by a 30-min incubation in tetramethylbenzidine. Absorbance was measured in a microplate reader at 450 nm and wavelength correction was applied at 540 nm.

### qRT-PCR

Total messenger RNA was extracted from bone-marrow-derived macrophages using Tri-Reagent Solution (Life technologies-T9424) and reverse transcribed using the high-capacity cDNA reverse transcription kit from Applied Biosystems (Thermofisher Scientific Baltics UAB). Quantitative RT-PCR analysis was performed with a QuantStudio 12K Flex Real-Time PCR System (Life technologies) using a standard cycler protocol for SYBR green (Bio-Rad). All samples were normalized to Gapdh (glyceraldehyde-3-phospate dehydrogenase), Hprt (Hypoxanthine–guanine phosphoribosyltransferase) and β-actin by geometric mean. The control samples served as a reference value of 1. All simples were run in duplicate, and quantification was performed using 2^−ΔΔCtT^ method. The following primers were used: Hprt (**Forward**: TCAGTCAACGGGGACATAAA, **Reverse**: GGGGCTGTACTGCTTAACCAG). Gapdh (**Forward :** AGGTCGGTGTGAACGGATTTG, **Reverse** : TGTAGACCATGTAGTTGAGGTCA) β-actin (**Forward** : AGGAAGGAAGGCTGGAAGAG, **Reverse** : TCCCTGGAGAAGAGCTACGA) Interleukin 6 (**Forward** : CCTTCTTGGGACTGATGCTGGTG, **Reverse** : AGGTCTGTTGGGAGTGGTATCCTC) Arginase 1 (**Forward** : CTCCAAGCCAAAGTCCTTAGAG, **Foward** : CTCCAAGCCAAAGTCCTTAGAG) Retnla (**Foward** : GGAGCTGTCATTAGGGACATCA, **Reverse** : TCCCAAGATCCACAGGCAAA) Chil3 (**Forward** : TCTGGGTACAAGATCCCTGAA, **Reverse** : TTTCTCCAGTGTAGCCATCCTT) TNFα (**Foward** : AGCCGATGGGTTGTACCTTG **Reverse** : GTGGGTGAGGAGCACGTAGTC) EGR2 (**Forward** : CCCTTTGACCAGATGAACGGAG, **Reverse :** AAGCTACTCGGATACGGGAGATC).

### Statistical analysis

Results of univariate analyses are presented as means ± SEM. Data were compared by one way analysis of variance (ANOVA) followed by the non-parametric Kruskal–Wallis test. *P* < 0.05 was considered statistically significant.

For multivariate analysis of macrophage mRNA expression, surface protein expression, and cytokine secretion, we used multi-blocks analysis (Regularized Generalized Canonical Correlation Analysis, RGCCA) as described^31^. Briefly, for each polarization experiment we combined data from flow cytometry, mRNA expression and ELISA assay in order to evaluate the impact of irradiation on multiple parameters (concurrently. We considered each type of data as a block (a modality). We then illustrated how relationships between the most relevant variables can be displayed in a common space. In the search of biomarkers associated with the irradiation dose rate we applied RGCCA to identify variables from the three blocks (flow cytometry, mRNA expression, and ELISA data. The between-block connection associated with this objective of analysis is presented in Fig. 2 with an additional connection between the superblock and the irradiation dose. A superblock is defined as the concatenation of all the blocks and the corresponding global components can be derived. The space spanned by the global components is viewed as a compromise space that integrates all the modalities, which is called a common space. In order to ensure comparability between variables and blocks, the data have to be preprocessed. To make variables comparable, we standardize our data (zero mean and unit variance) and we opt for a strategy that divides each block by the square root of its number of variables^32^.

## Results

### LMDIR enhances mRNA expression of M2 cell markers in M0 and M2 type macrophages

**Figure 1:**
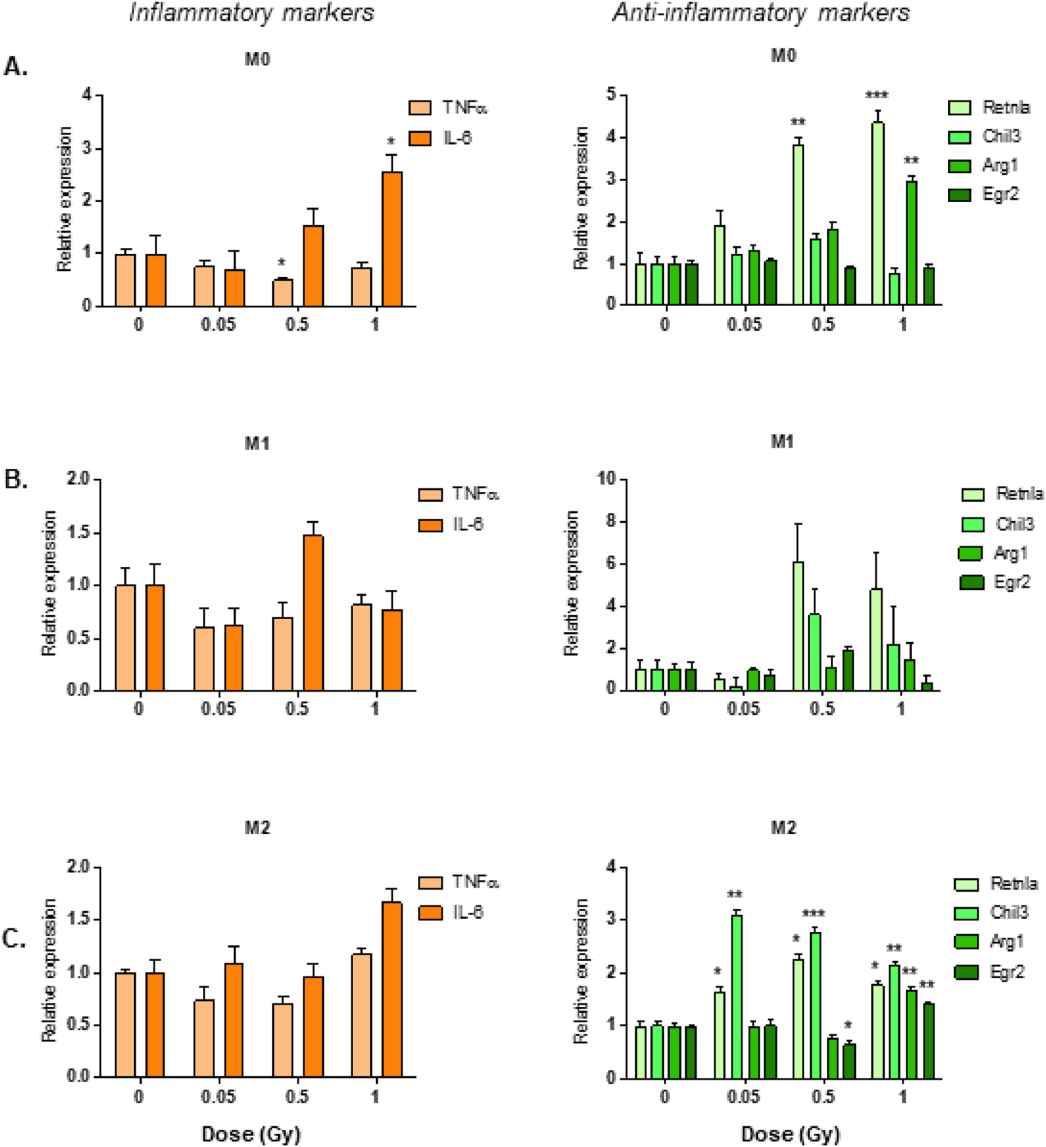
Exposure to LMDIR enhances anti-inflammatory markers in M0 and M2 macrophages derived from cells obtained 1 day post-irradiation. ApoE^(−/−)^ mice were exposed to 0, 0.05, 0.5 or 1 Gy of irradiation. Bone marrow cells were extracted 1 day later and placed 5 days in culture with M-CSF. Resulting macrophages were maintained as M0 (**A**) or polarized into M1 (**B**) or M2 (**C**) macrophages. Gene expression of pro-inflammatory (IL-6, TNFα) or anti-inflammatory (Retnla, Chil3, Arg1, Egr2) markers was determined by qRT-PCR. Results are expressed as fold change vs BMDM from non-irradiated control mice. Data are mean ± SEM of n = 3 to 4. *p < 0.05, **p < 0.01, ***p < 0.001.

As previously discussed, low doses of ionizing irradiation have a modulatory effect on inflammation. Here we studied macrophage secretory function to provide additional insight on the impact of LMDIR. Bone marrow cells were obtained ApoE^(−/−)^ mice 1 day after the animals were exposed to different doses of irradiation. The cells were kept in culture for 5 days with M-CSF, then they were polarized with IFNγ or IL-4 to yield M1 or M2 type macrophages, respectively. Unpolarized macrophages remained as M0. Figure 1 represents levels of mRNA expression of pro-inflammatory M1 markers IL-6 and TNF-α and anti-inflammatory M2-markers Arginase 1, Retnla, Chil3, and Egr2 in M0, M1, and M2 macrophages. Irradiation had little effect on M1 marker expression. In M0 macrophages, we observed that TNF-α expression was reduced by 50% subsequent to 0.5 Gy exposure, whereas IL-6 was more than doubled following 1 Gy exposure, compared with M0 cells from non-irradiated mice (*p* = 0.05) (Fig. 1A). No such effects were observed in M1 or M2 polarized cells. On the contrary, M2 markers tended to be upregulated in all macrophages derived from irradiated mice compared with non-irradiated animals, reaching significance in M0 and M2 cells. Maximum exposure (1 Gy) upregulated Retnla (4.5-fold, *p* = 0.0005) and Arg1 (2.9-fold, *p* = 0.002) mRNA in M0 macrophages (Fig. 1A), whereas it significantly (*p* < 0.05) enhanced the expression of all M2 markers in M2-polarized cells (Fig. 1C). Even at doses as low as 0.05 Gy, irradiation led to increased mRNA expression of Retnla (1.66-fold, *p* = 0.04) and Chil3 (3.1-fold, *p* = 0.005) (Fig. 1C). These results suggest that low dose irradiation of mice promotes a generally anti-inflammatory phenotype in BMDM.

### LMDIR enhances pro- and anti-inflammatory cytokine secretion by macrophages

**Figure 2:**
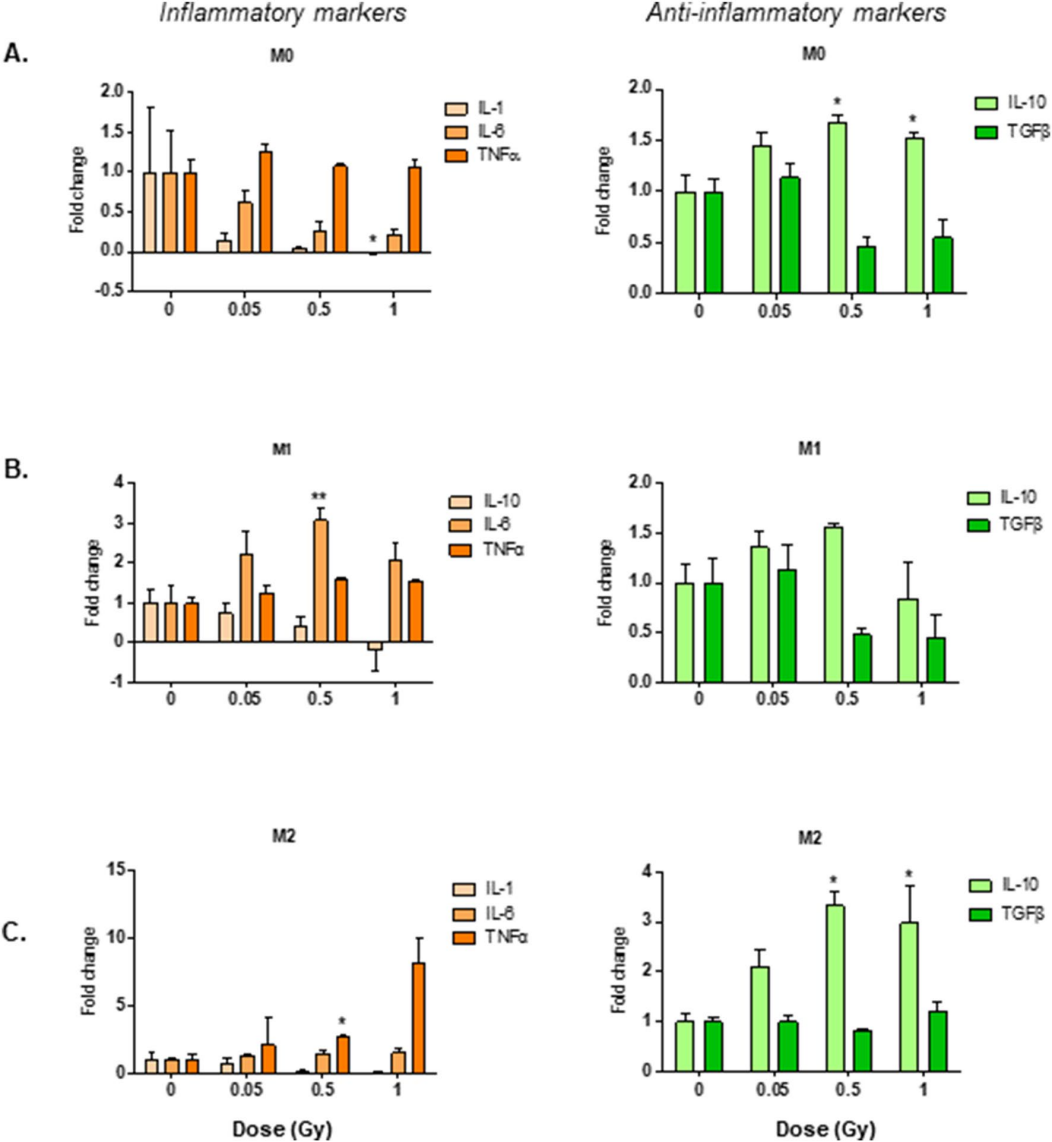
Exposure to LMDIR enhances anti-inflammatory IL-10 secretion in M0 and M2 macrophages derived from cells obtained 1 day post-irradiation. ApoE^(−/−)^ mice were exposed to 0, 0.05, 0.5 or 1 Gy of irradiation. Bone marrow cells were extracted 1 day later and placed 5 days in culture with M-CSF. Resulting macrophages were maintained as M0 (**A**) or polarized into M1 (**B**) or M2 (**C**) macrophages. Secretion of pro-inflammatory (IL-1, IL-6, TNFα) and anti-inflammatory (IL-10, TGFβ) cytokines in the conditioned medium of these cells was measured by ELISA. Results are expressed as fold change vs BMDM from non-irradiated control mice. Data are mean ± SEM of n = 3 to 4. *p < 0.05, **p < 0.01, ***p < 0.001.

In parallel with the mRNA expression experiments described above, we evaluated the cytokines released during the last 24 h of culture of polarized or unpolarized macrophages, comparing cells derived from non-irradiated mice with those from mice receiving different doses of irradiation 1 day before extraction. In M0 cells derived from mice exposed to LMDIR, we observed a shift towards an anti-inflammatory secretome characterized by complete loss of IL-1β following 1 Gy and ≥ 1.5-fold enhancement of IL-10 following 0.5 or 1 Gy (*p* < 0.05) (Fig. 2A). Effects in M1 cell were more subdued, showing a threefold increase of IL-6 at 0.5 Gy only (*p* = 0.008) (Fig. 2B). Finally, we detected an increase in the secretion of IL-10 by M2 macrophages at all doses tested: 2.1-fold at 0.05 Gy (*p* = 0.017), 3.3-fold at 0.5 Gy (*p* < 0.01), and threefold at 1 Gy (*p* = 0.036) (Fig. 2A–C).

### Superblock analysis correlates lL-10 secretion with LMDIR in M0 and M2 macrophages

**Figure 3:**
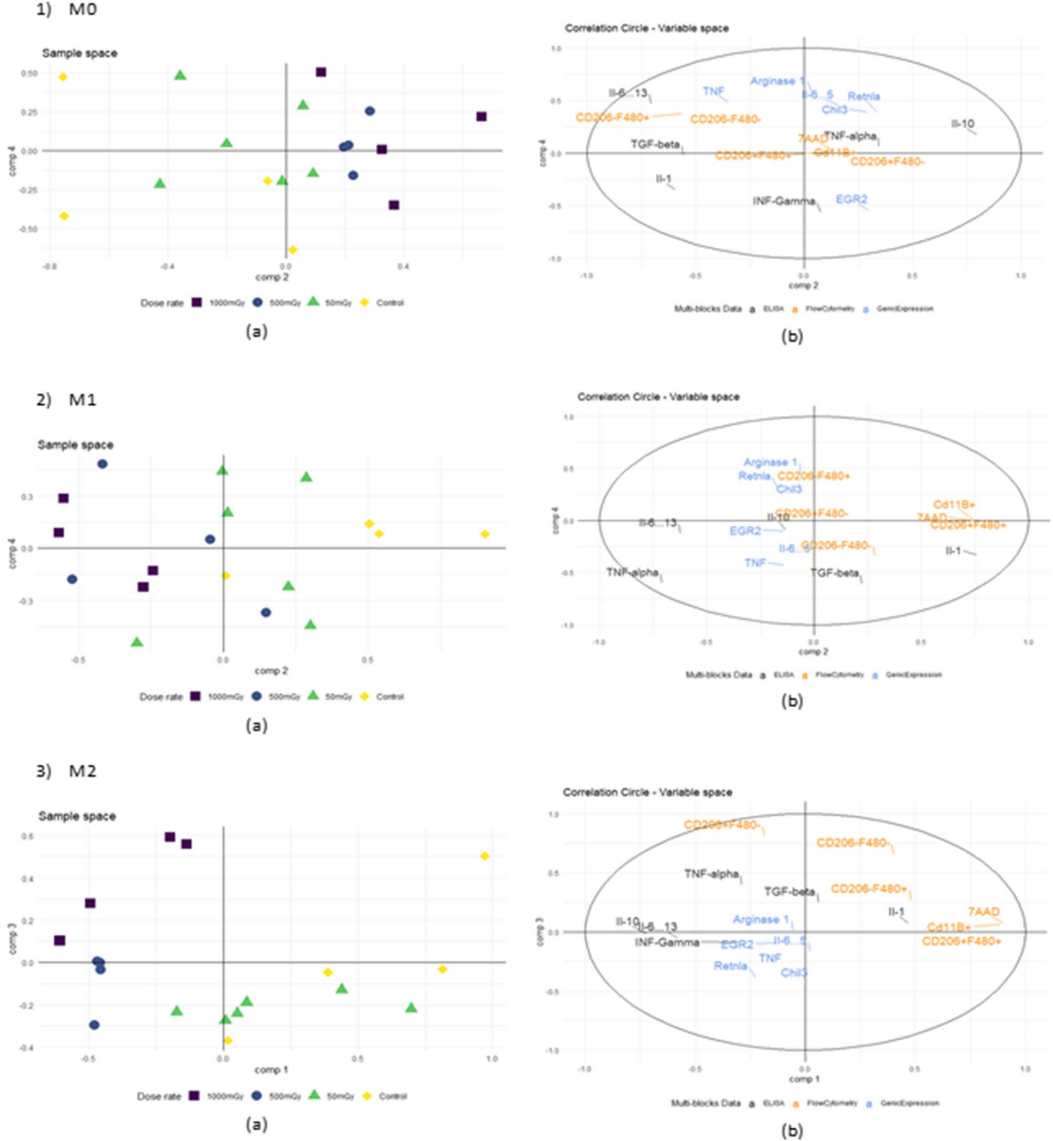
Effects of LMDIR on gene expression, cytokine secretion, and immune phenotype of macrophages derived from bone marrow cells obtained 1 day post-irradiation*.* ApoE^(−/−)^ mice were exposed to 0, 0.05, 0.5 or 1 Gy of irradiation. Bone marrow cells were extracted 1 day later and placed 5 days in culture with M-CSF. Resulting macrophages were maintained as M0 (**1**) or polarized into M1 (**2**) or M2 (**3**) macrophages. Results show (**a**) sample space associated with principal dimensions of the superblock principal according to irradiation dose and (**b**) RGCCA variable space analysis of supernatant cytokine concentration (black), cell mRNA expression (blue), and cell marker protein expression (orange).

We undertook flow cytometry analysis of the macrophages derived from bone marrow cells obtained 1 day post-exposure. No notable differences in phenotype were observed between cells from irradiated or non-irradiated mice (data not shown). However, those data provided interesting insight when integrated in the RGCCA analysis along with input from qRT-PCR and ELISA assays. There was a clear separation according to component 1 among M0 macrophages between lower doses (control and 0.05 Gy groups) and higher doses (0.5 and 1 Gy groups) of irradiation (Fig. 3-1). The separation of lower doses corresponded to IL-1β secretion whereas the higher doses were associated with IL-10 cytokine secretion and with F4/80-CD206+ cell phenotype. Interestingly, we observed a more generalized pro-inflammatory secretion in M1 macrophages, as IL-1β was once again significantly associated with lower doses of irradiation and TNFα correlated with higher doses (Fig. 3-2). Finally, M2 type macrophages had a response similar to M0 type macrophages, where lower doses favoured an enhancement of IL-1β secretion, and higher doses correlated significantly with elevated IL-10 (Fig. 3-3).

### Changes in circulating and spleen Ly6C^High^ and Ly6C^Low^ monocytes post irradiation

In a separate set of experiments, we analyzed blood and spleen cells 1, 10, or 100 days post-irradiation by flow cytometry. We compared pro-inflammatory Ly6C^High^ monocytes which tend to accumulate in atherosclerotic plaques to patrolling, anti-inflammatory Ly6C^Low^ monocytes)^19^.

**Figure 4:**
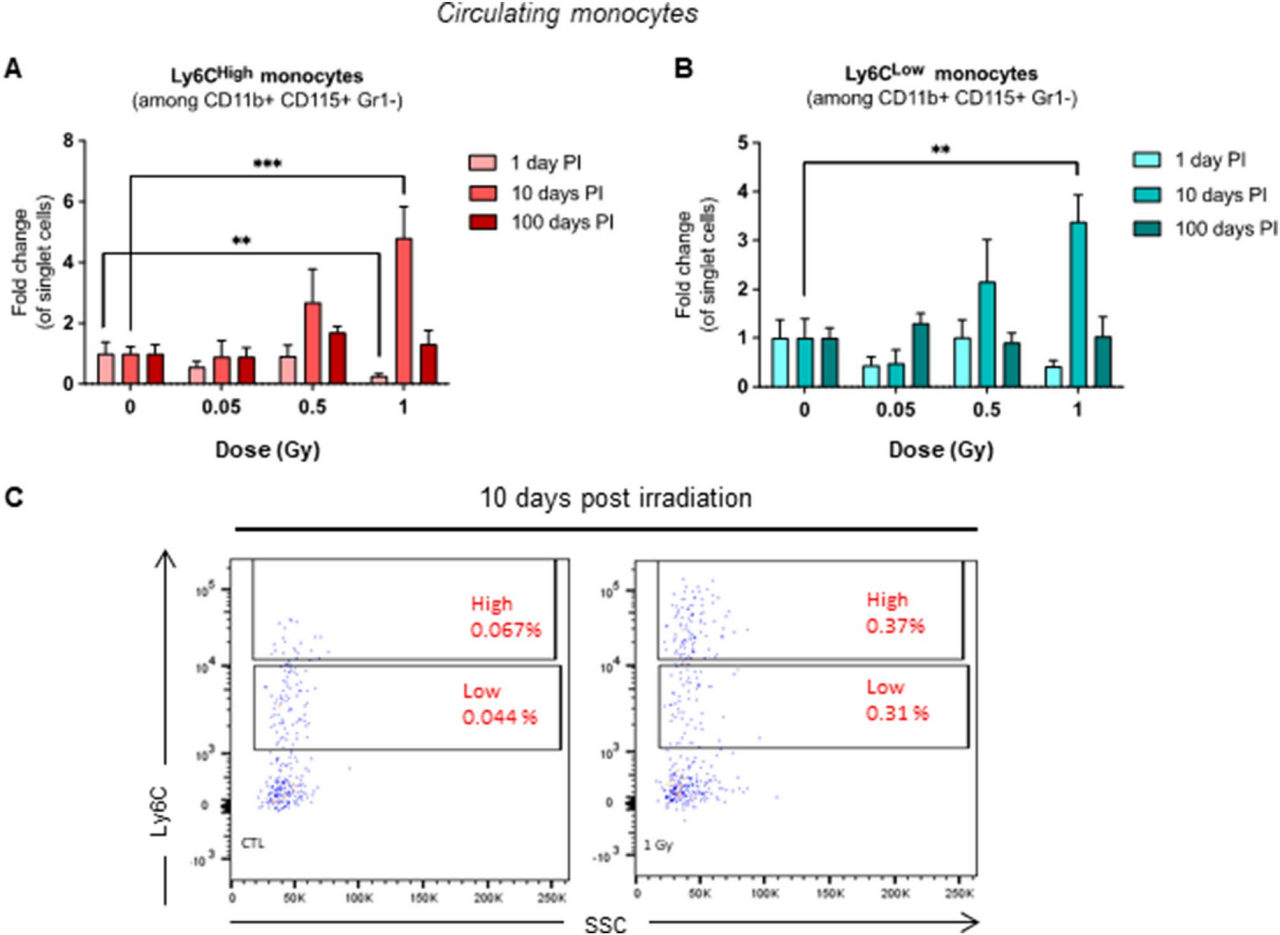
Proportions of Ly6C^High^ monocytes in blood are decreased 1 day and increased 10 days after exposure to LMDIR. ApoE^(−/−)^ mice were exposed to 0, 0.05, 0.5 or 1 Gy of irradiation. Blood was extracted by cardiac puncture 1, 10, or 100 days post-irradiation. Proportions of Ly6C^High^ (**A**) or Ly6C^Low^ (**B**) monocytes were evaluated by flow cytometry. **C:** Representative dot plot analysis of cells obtained from a non-irradiated (left) and an irradiated mouse (right) 10 days post-exposure at 1 Gy. Results are expressed as fold change proportion of Ly6C^High^ or Ly6C^Low^ monocytes among single cells, comparing data from irradiated mice with that of non-irradiated control mice (CTL, set as a value of 1). Data are mean ± SEM of n = 5 to 8. **p < 0.01, ***p < 0.001.

Only the high dose (1 Gy) of irradiation affected circulating monocyte populations. Ly6C^High^ monocytes were significantly decreased 1 day post-irradiation, to 0.25-fold of non-irradiated control (*p* = 0.002). At 10 days post-irradiation, the opposite effect was observed: Ly6C^High^ monocytes were increased threefold (*p* < 0.001). Then again, Ly6C^Low^ monocyte numbers were also increased at that time, reaching a fivefold increase (*p* = 0.004). Finally, after 100 days, effects of irradiation were no longer observed (Fig. 4A). No significant changes occurred at any other time points or doses (Fig. 4B,C).

**Figure 5:**
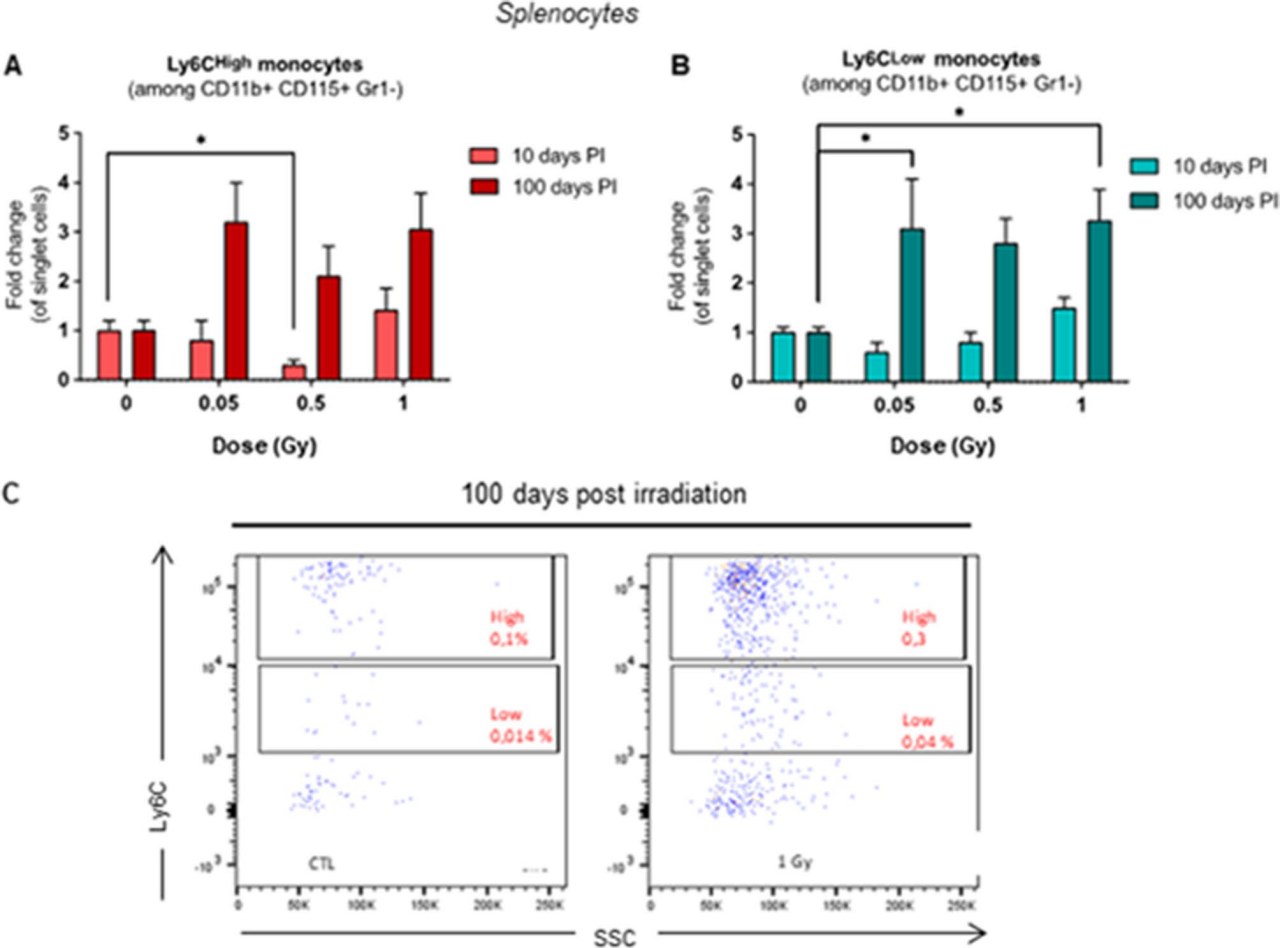
Proportions of Ly6C^Low^ monocytes in the spleen are enhanced 100 days after exposure to LMDIR. ApoE^(−/−)^ mice were exposed to 0, 0.05, 0.5 or 1 Gy of irradiation. Spleens were obtained 1, 10, or 100 days post-irradiation and splenocytes evaluated by flow cytometry, distinguishing Ly6C^High^ (**A**) or Ly6C^Low^ (**B**) monocyte populations. (**C**) Representative dot plot analysis of cells obtained from a non-irradiated (left) and an irradiated mouse (right) 100 days post-exposure at 1 Gy. Results are expressed as fold change proportion of Ly6C^High^ or Ly6C^Low^ monocytes among single cells, comparing data from irradiated mice with that of non-irradiated control mice (CTL, set as a value of 1). Data are mean ± SEM of n = 5 to 8. **p < 0.01, *p < 0.05.

The spleen can be a site of extramedullary hematopoiesis. It has been shown to be a monocyte reservoir^33^ and is believed to be the source of inflammatory Ly6C^High^ monocytes that can be mobilize to injured vessels and infiltrate atherosclerotic lesions^21^. Flow cytometry analysis of spleen samples provided an insight of Ly6C^High^ and Ly6C^Low^ proportions at two time points, 10 and 100 days post-irradiation. At day 10, Ly6C^High^ monocytes were reduced threefold (*p* = 0.015) in the spleens of mice exposed to 0.5 Gy compared with the non-irradiated control group (Fig. 5A). On the contrary, we observed a significant threefold increase in Ly6C^Low^ monocytes at 100 days post-irradiation at 0.05 (*p* = 0.048) and 1 Gy (*p* = 0.035) (Fig. 5B,C).

### LMDIR has little impact on atherosclerotic plaque size and composition 100 days post-irradiation

**Figure 6:**
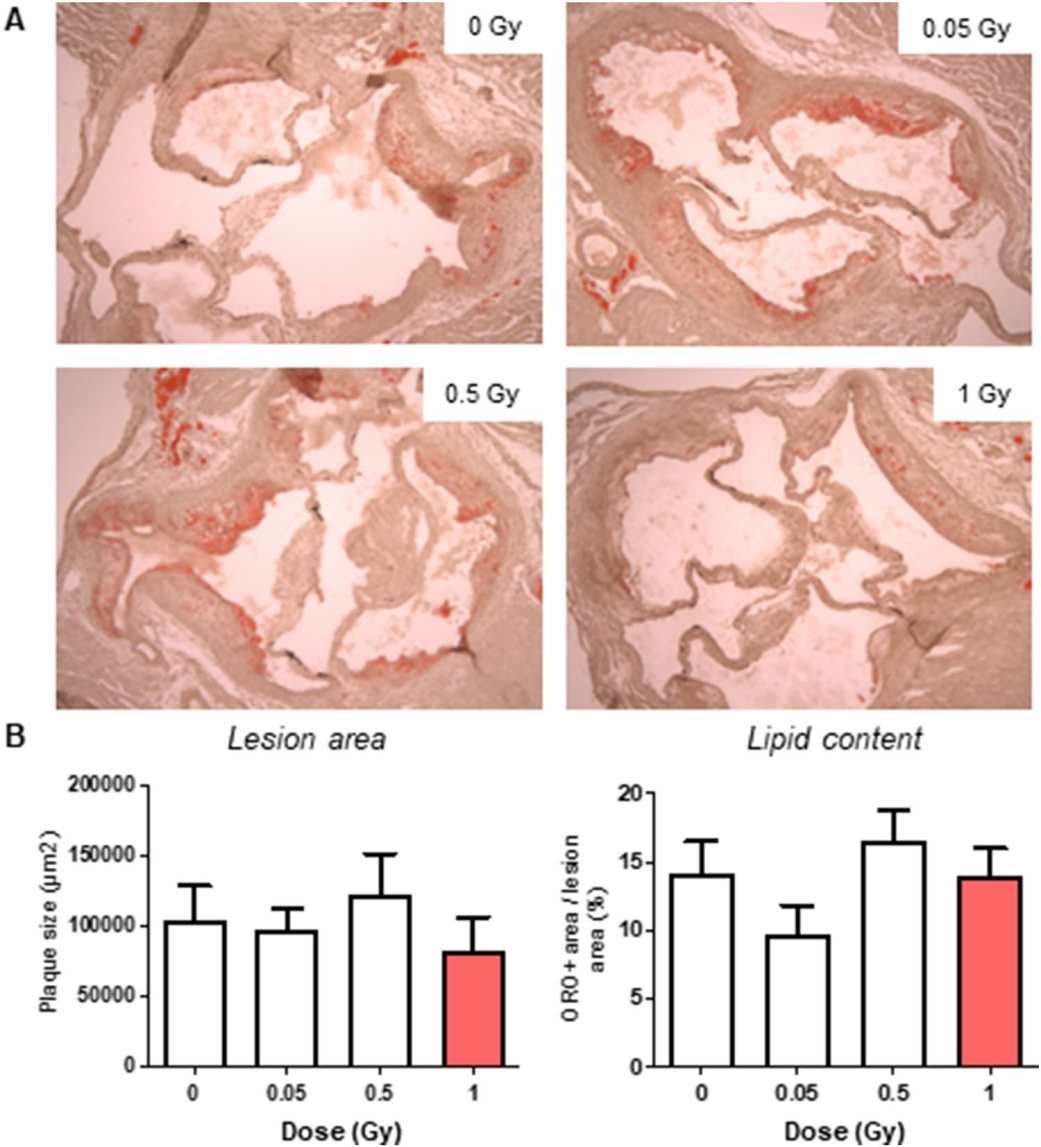
Atherosclerotic plaque size and lipid content are not altered 100 days after exposure to LMDIR. ApoE^(−/−)^ mice were exposed to 0, 0.05, 0.5 or 1 Gy of irradiation. Atherosclerotic lesions developing in the aortic sinus were analyzed 100 days later. Oil red O staining was performed on cryosections. (**A)** Representative images obtained at ×100 magnification. (**B)** Quantification of positive staining within the plaque area. Data are mean ± SEM of n = 5 to 6 animals. Five sections were analyzed for each animal.

**Figure 7:**
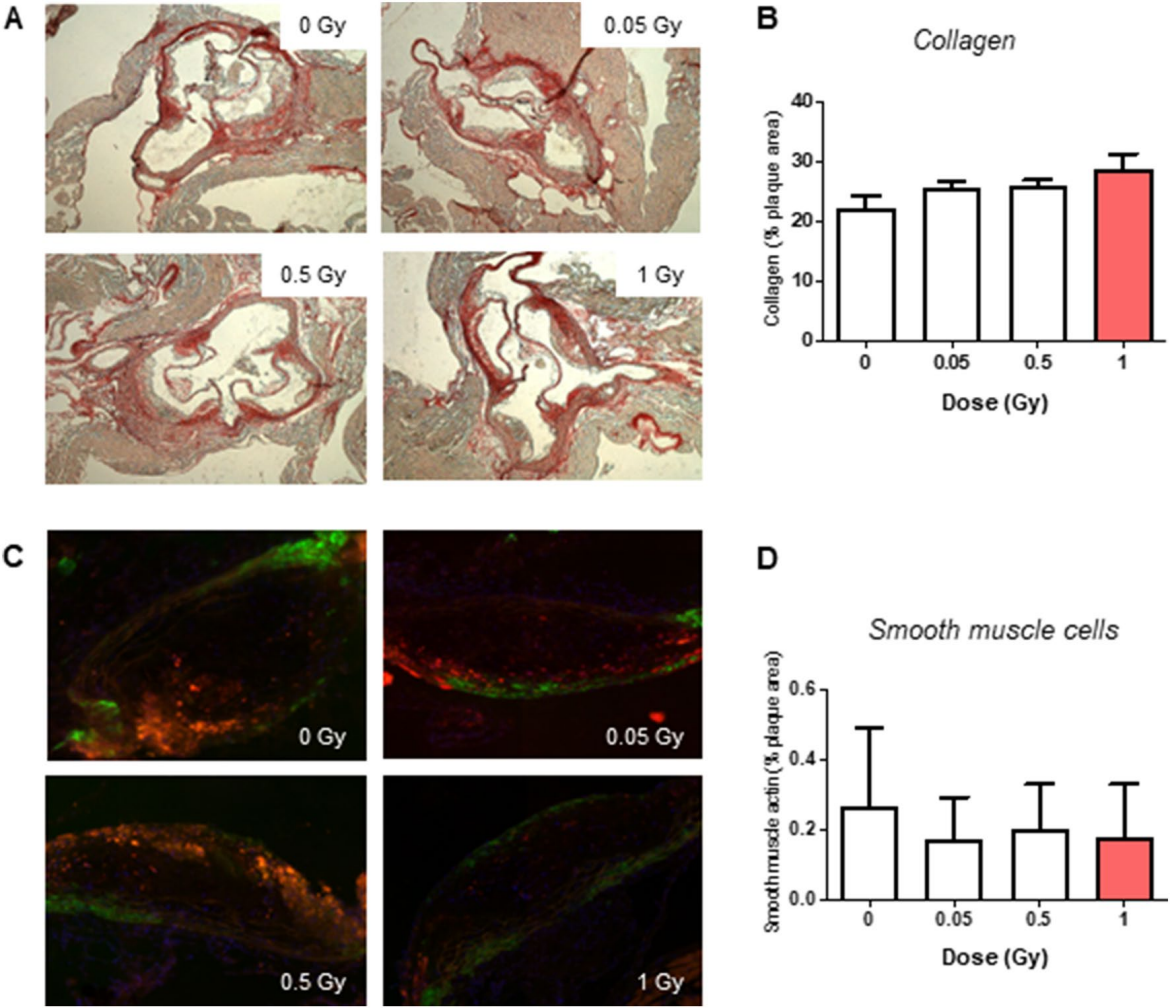
Atherosclerotic plaque collagen and smooth muscle cell content are not altered 100 days after exposure to LMDIR. ApoE^(−/−)^ mice were exposed to 0, 0.05, 0.5 or 1 Gy of irradiation. Atherosclerotic lesions developing in the aortic sinus were analyzed 100 days later. (**A)** Picrosirius red staining for collagen was performed on cryosections. Representative images obtained at ×50 magnification. (**B)** Quantification of collagen staining within the plaque area. (**C)** SMCs were detected by α-SMA immunostaining (green). Representative images were obtained at ×100 magnification. (**D)** Quantification of α-SMA staining within the plaque area. Data are mean ± SEM of n = 4–6 animals. Five to 8 sections were analyzed for each animal.

**Figure 8:**
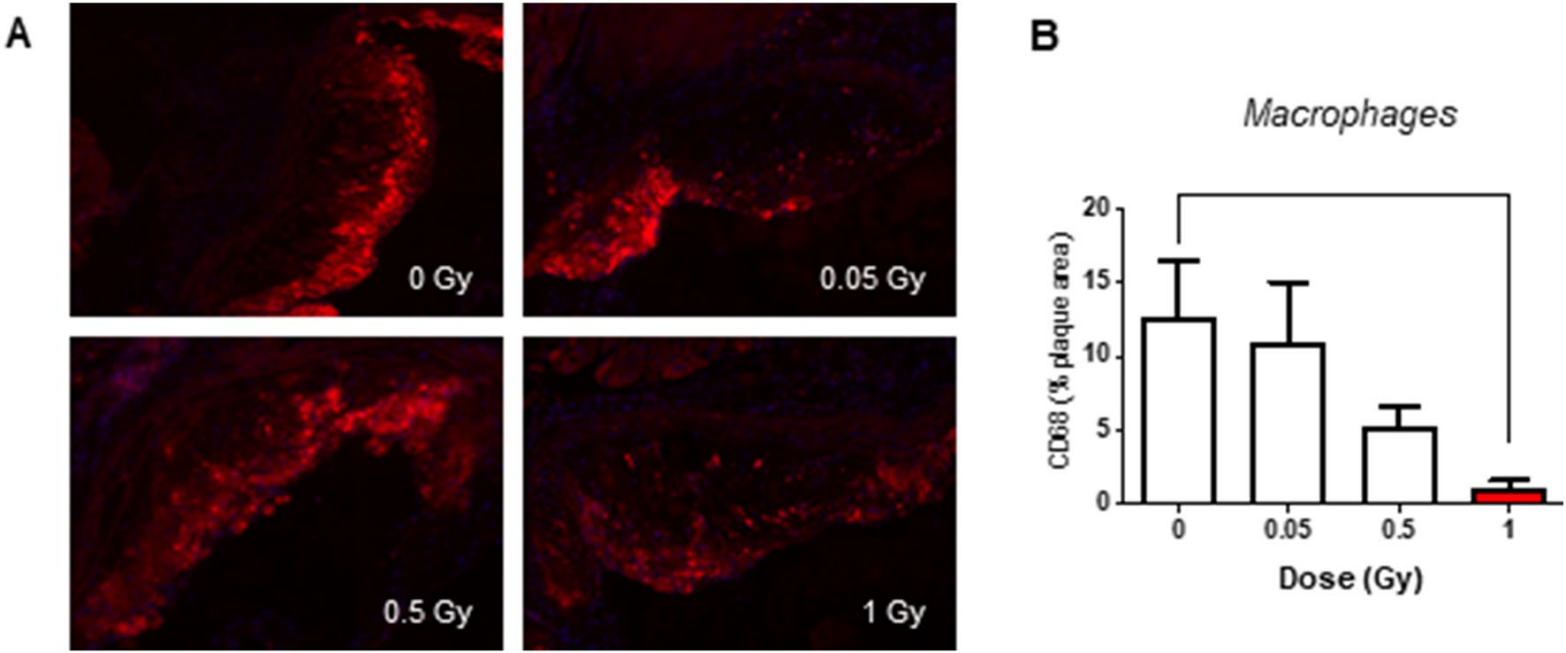
Atherosclerotic plaque macrophage content is reduced 100 days after exposure to LMDIR at 1 Gy. ApoE^(−/−)^ mice were exposed to 0, 0.05, 0.5 or 1 Gy of irradiation. Atherosclerotic lesions developing in the aortic sinus were analyzed 100 days later. (**A)** CD68 immunostaining for macrophages was performed on cryosections. Representative images obtained at ×100 magnification. (**B)** Quantification of CD68+ staining within the plaque area. Data are mean ± SEM of n = 4 to 6 animals. Five to 8 sections were analyzed for each animal. *p < 0.05.

**Figure 9:**
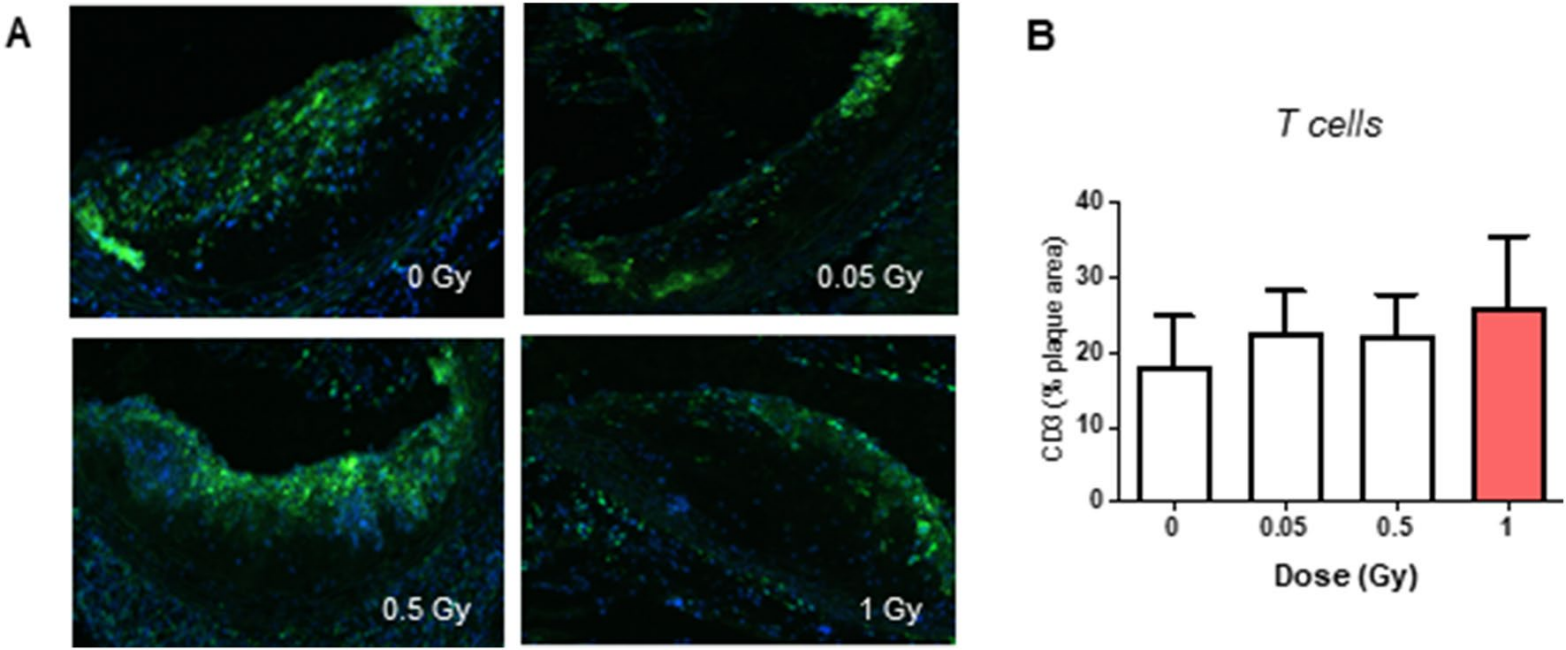
Atherosclerotic plaque T cell content is unchanged 100 days after exposure to LMDIR. ApoE^(−/−)^ mice were exposed to 0, 0.05, 0.5 or 1 Gy of irradiation. Atherosclerotic lesions developing in the aortic sinus were analyzed 100 days later. (**A**) CD3 immunostaining for T cells was performed on cryosections. Representative images obtained at ×100 magnification.**B** Quantification of CD3+ staining within the plaque area. Data are mean ± SEM of n = 5 to 6 animals. Five to 8 sections were analyzed for each animal.

Finally, we evaluated the atherosclerotic lesions of mice 100 days after their exposure to different doses of irradiation. Assessment of plaque size and composition using Oil red O staining revealed no differences in atherosclerotic lesion size or lipid content (Fig. 6A–D). Similarly, neither collagen content, assessed by Picrosirius red staining (Fig. 7A,B), nor α SMA content (Fig. 7C,D) showed any differences at any dose of exposure. However, plaque CD68+ macrophage foam cell content was found to decrease gradually with rising doses of irradiation, covering 12.5% of lesion area in non-irradiated mice but only 1.5% in mice exposed to 1 Gy (*p* = 0.036) (Fig. 8A,B). Finally, plaque lymphocyte content, assessed by CD3 staining (Fig. 9A,B), did not differ between groups.

## Discussion

In this study, we evaluated short- and long-term effects of LMDIR on monocytes and macrophages in atheroprone ApoE^(−/−)^ mice. We found that moderate doses of irradiation favor anti-inflammatory Ly6C^Low^ monocytes and promote M2 phenotype skewing accompanied by the release of the anti-inflammatory cytokine IL-10 by M0 and M2 macrophages. Additionally, LMDIR triggers a decrease in plaque CD68+ macrophages that reaches significance at 1 Gy.

Macrophages can be categorized as classically activated M1 type that mediate host defense against a wide range of pathogens and participate in several chronic inflammatory diseases including atherosclerosis^19,34^, or they can be defined as alternatively activated M2 type that secrete anti-inflammatory IL-10 and TGFβ and are involved in wound healing^25,33–35^. Our study shows that LMDIR led to the expression of a genetic signature typical of M2 macrophages, such as Egr2, Arg-1, Chil3 and Retnla^36^. These genes were all upregulated subsequent to the 1 Gy dose and Chil3 and Retlna were also significantly upregulated at lower doses (0.05 and 0.5 Gy). IL-4, one of the main cytokines driving M2 polarization, is an enhancer of Arg-1, Chil3, Retnla, and Egr2^37,38^. Hence, LMDIR may facilitate the role of IL-4 in promoting the expression of these specific genes. Also, since it has been shown that knock-down of Egr2 decreases the expression of Arg-1, Retnla, and Chil-3^27^, the enhanced expression of Egr2 could stimulate expression of the other three M2 markers.

Exposure to LMDIR at 0.5 or 1 Gy also enhanced the secretion of IL-10 by M0 and M2 macrophages, suggesting that moderate doses of irradiation potentiate the anti-inflammatory role of these cells. Concurrently, RGCCA analysis correlated the enhanced secretion of IL-10 with higher proportions of CD11b+F4/80-CD206+ macrophages. CD206 is a transmembrane glycoprotein considered as an M2 type macrophage marker^27,38,39^ and is known to be upregulated by IL-10^40^. One could posit that LMDIR enhances anti-inflammatory IL-10 secretion, leading to all observed phenotypic changes in macrophages. However, there is no evidence in the literature that IL-10 stimulates the expression of M2 marker genes upregulated by LMDIR in our study. In addition, although Egr2 could stimulate the expression of Arg-1, Retnla, and Chil-3, it is known to inhibit IL-10^37^, excluding its potential role as a master regulator. Therefore, LMDIR is likely to impact both cytokine secretion and gene expression independently.

In another study, bone marrow-derived macrophages obtained from irradiated C57BL/6 mice were also found to have an anti-inflammatory profile, with enhanced gene expression of M2 markers such as Arg-1, Chi3l3, and Ym1/2^40^. These effects were obtained with much higher dose than that used by us (4 Gy) but outcomes were surprisingly similar. More recently, Wunderlich et al.^41^ exposed peritoneal macrophages to a low dose of X rays (0.5 Gy), and found that M1 type macrophages secreted higher levels of TGF-β and lower levels of IL-1β. Which is consistent with an anti-inflammatory response. In the context of atherosclerosis, macrophage polarization towards an M2 phenotype could have a potential protective effect on the disease, by reducing inflammation.

Regarding blood monocytes, the Ly6C^HIgh^ population was greatly reduced by 1 Gy exposure after 1 day, but the opposite effect was observed at day 10 post-irradiation, when a fourfold increase compared to the control group was observed. Similarly, patrolling Ly6C^low^ monocytes were increased 10 days after 1 Gy exposure. This suggests that 1 Gy irradiation may produce a depletory effect on monocytes at day 1, followed by reactive proliferation a few days thereafter. Given that both monocyte subsets followed the same trend, it is unlikely that irradiation induced monocyte conversion from Ly6C^Hiigh^ to Ly6C^Low^. Changes in monocyte numbers were no longer observed at day 100 post irradiation.

Interestingly, splenic Ly6C^Low^ monocyte numbers were significantly increased at 100 days post-irradiation rather than 10 days for the circulating monocytes, and at a broader range of irradiation doses. This profile indicates a prolonged effect of LMDIR on the spleen Ly6C^Low^ reservoir. One possible explanation for this could be that increased blood Ly6C+ /− monocytes enter the spleen over time and accumulate there preferentially. We know that the spleen can be an important source of monocytes during inflammation and that monocytes can be recruited from there into the atherosclerotic plaque^33^. Splenocytes are also important in resolving inflammation in heart failure^42^. The use of fate mapping techniques or adoptive transfer could help to predict how the effects of LMDIR on the splenic reservoir impact the atherosclerotic plaque.

One of the main questions of our study was long term effects of LMDIR on plaque size, stability, and inflammatory profile. Atherosclerotic plaques are characterized by the proliferation of oxidized LDL-capturing macrophages, responsible for the formation of a highly inflammatory necrotic core. This phenomenon is associated with plaque instability. Reduced vascular smooth muscle cell and collagen contents, especially in the context of a thin fibrous cap, further characterize lesions prone to rupture^43^. Although plaque rupture is rare in mice compared with humans, morphological features of the atheroma are comparable between the species^44^. We found that neither plaque collagen nor smooth muscle content showed differences between irradiated and non-irradiated animals. Likewise, LMDIR did not affect plaque size or lipid contents. However, although CD3+ lymphocytes were unchanged within lesions, CD68+ macrophages were significantly decreased by 1 Gy exposure, suggesting that moderate irradiation exposure decreases inflammation or reduces macrophage proliferation in atheromatous plaques. Correspondingly, LMDIR enhanced the M2 skewing and anti-inflammatory function of macrophages in vitro. An increased IL-10 secretion inhibits macrophage activation and proliferation^45^ and M2 gene markers are suggestive of reparative macrophages associated with plaque regression. Hence the decrease in total plaque macrophages 100 days post irradiation could be a long-term consequence of the M2 macrophage skewing by LMDIR. It is worth noting that the CD68 marker could also be expressed by foamy cells of smooth muscle origin^46^. Nevertheless, whatever their origin, CD68+ cells would be expected to contribute to the inflammatory state of the plaque, such that reduced proportions in LMDIR support improved lesion stability.

In summary, our results suggests that exposure of ApoE^(−/−)^ mice to LMDIR regulates monocyte and macrophage responses related to the development of atherosclerosis. Although irradiation was associated with increased numbers of circulating monocytes, preferential accumulation of patrolling Ly6C^Low^ monocytes in the spleen and skewing of in vitro macrophages towards an M2 phenotype secreting the anti-inflammatory cytokine IL-10 were deemed to be indicative of an anti-inflammatory profile. Despite modest effects on plaque size and composition, LMDIR may have a beneficial impact by reducing lesion CD68+ cell content. These findings are in line with and further explain the atheroprotective effects of moderate doses of ionizing radiations observed in previous studies^10–13^.
